# An Eco‐Friendly, Tunable and Scalable Method for Producing Porous Functional Nanomaterials Designed Using Molecular Interactions

**DOI:** 10.1002/cssc.201700027

**Published:** 2017-03-29

**Authors:** Joseph R. H. Manning, Thomas W. S. Yip, Alessia Centi, Miguel Jorge, Siddharth V. Patwardhan

**Affiliations:** ^1^ Department of Chemical and Biological EngineeringUniversity of SheffieldMappin StreetSheffieldS1 3JDEngland; ^2^ Department of Chemical and Process EngineeringUniversity of Strathclyde75 Montrose StreetGlasgowG1 1XJScotland

**Keywords:** green nanomaterials, manufacturing, molecular interactions, porous materials, silica

## Abstract

Despite significant improvements in the synthesis of templated silica materials, post‐synthesis purification remains highly expensive and renders the materials industrially unviable. In this study this issue is addressed for porous bioinspired silica by developing a rapid room‐temperature solution method for complete extraction of organic additives. Using elemental analysis and N_2_ and CO_2_ adsorption, the ability to both purify and controllably tailor the composition, porosity and surface chemistry of bioinspired silica in a single step is demonstrated. For the first time the extraction is modelled using molecular dynamics, revealing that the removal mechanism is dominated by surface‐charge interactions. This is extended to other additive chemistry, leading to a wider applicability of the method to other materials. Finally the environmental benefits of the new method are estimated and compared with previous purification techniques, demonstrating significant improvements in sustainability.

## Introduction

Porous nanomaterials are of wide interest to academia and industry owing to their diverse range of available pore systems, and when functionalised, they form a versatile platform for applications such as catalysis, separation, drug delivery, sensors and biomedical implants.^1^, ^2^ Templated silica (e.g., MCM‐41 or SBA‐15) in particular combines chemical and physical versatility with high starting‐material availability, leading to a wide variety of morphologies and functionalities tailored for specific applications.^3^

However, there are marked environmental issues with the production of templated silicas using current methods, as demonstrated by E‐factor and other analyses in recent reviews.^4^, ^5^, ^6^, ^7^ These production methods subdivide into three synthesis steps: initial templated materials synthesis; removal of the organic template; and an optional but common chemical functionalisation post‐purification.^7^ Many issues are present in these methods at all stages of the synthesis,^4^, ^8^ ranging from the necessity for autoclave conditions during synthesis to energy‐inefficient and destructive methods of template removal and to the use of hazardous and moisture‐sensitive organosilanes to obtain chemical functionality. This leads to uneconomical and environmentally damaging materials production, thus preventing industrial implementation.

Work in our group and by others has previously attempted to reduce waste in the synthesis step by using alternative, bioinspired organic “additives” to the norm.^9^, ^10^ Studies on these bioinspired silicas have shown that they have equal or better performance in some applications than MCM‐41,^11^, ^12^ whereas the cost of synthesis has been significantly reduced compared to that of bulk precipitated silicas.^13^

However, this has addressed only one part of the production process. In all of these studies, templates were removed by destructive calcination methods and no other alternatives have been reported. In contrast, numerous studies have been performed on conventional templated materials to remove the template in a non‐destructive manner using solvent extraction.^4^ However, to date, complete removal of templates from MCM‐41 or SBA‐15 through solvent extraction has not been possible.^5^, ^14^ This is caused by the large energetic driving force required to break the organic–inorganic interfacial interaction. Therefore, several studies have used microwave^15^ or other irradiation^16^ and supercritical^17^ or refluxing solvents^18^, ^19^ to achieve complete extraction of templates and template recycling after elution.^20^ Although there have been reports of complete template elution without the need for such promoters, these require that the material is designed to allow for elution through the choice of template molecule, and therefore compromise materials properties compared to their parent materials.^4^, ^19^ For example, hexagonal‐mesoporous silica (HMS) uses dodecylamine (DDA) templates rather than cetyltrimethylammonium (CTA for MCM‐41) but has lower ordered pore domains than MCM‐41 as a result.^19^, ^21^ Further, although greatly improved by their non‐destructive nature, such extraction methods require reflux (high temperature) in alcohol or acidified water for at least 1 h. Hence, these methods have high energy demands (more than calcination, see the Results and Discussion section) leading to prohibitive costs for industrial implementation. As such, significant advances are required to the solvent‐elution methods to reduce the large environmental costs of purification.

Herein, we attempt to apply the strategy of solvent elution rather than calcination to the production of bioinspired silica. We demonstrate a new, acid‐based, room temperature additive elution method to purify bioinspired silica in a single, rapid, post‐synthetic step (something that has not previously been reported for bioinspired or mesoporous silica). Furthermore, by controlling the pH value of bioinspired silica suspensions, controllable partial elution of the organic additive is achieved, thus directly functionalising the material in a single step. This both reduces the synthesis complexity and obviates the need for hazardous organosilane reagents, which are commonly required for similar surface modifications.

For the first time in such a study, we have applied detailed molecular dynamics (MD) simulations of the organic–inorganic interface to model the non‐covalent interactions between organic additive and silica surface. Using this, we compare the results from our study to previous literature examples of solvent elution and rationalise the differences in required purification driving forces against interaction strength and template hydrophobicity. We therefore propose a general strategy for developing mild solvent elution using MD to predict template‐extraction efficiency.

Finally, we perform a preliminary techno‐economic analysis of our new method to estimate and compare the environmental and economic savings of using our acid‐elution method over conventional calcination or solvent‐elution techniques.

## Results and Discussion

### Acid treatment as a purification method

**Figure 1:**
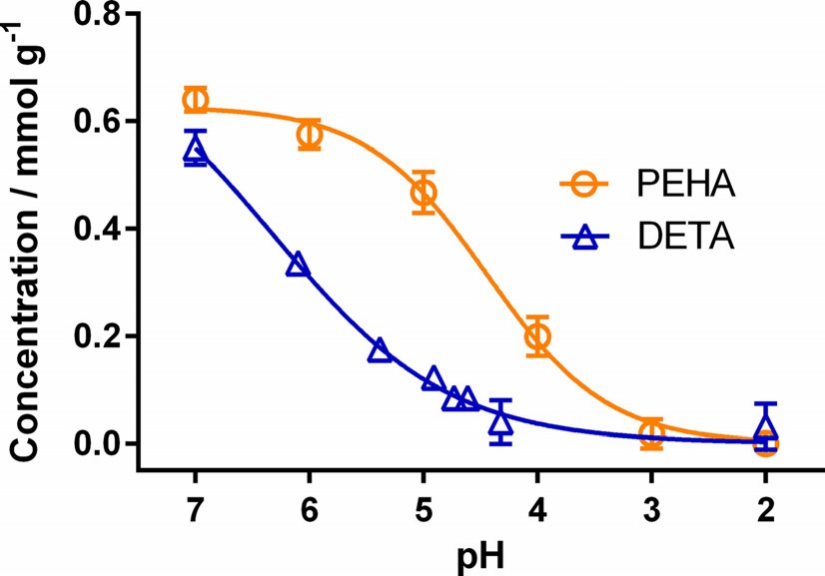
Graph of additive concentration in silica versus acidification pH value determined by elemental analysis for both PEHA and DETA.

Bioinspired silica was synthesised using pentaethylenehexamine (PEHA) as an additive owing to its high catalytic activity.^22^ Once synthesised at pH 7, the suspension was treated with acid for 10 min to reach a desired pH value between 7 and 2 (the isoelectric point of silica).^23^ Upon such treatment, the concentration of PEHA in silica was found to decrease (Figure 1), indicating additive removal. The removal was found to be proportional to the pH value in a nonlinear fashion: treatment to pH≥5 was found to have a small effect on additive content (ca. 25 % additive removed); however, after further treatment to pH 4, the majority of additive (ca. 70 %) had been eluted. Acidification to pH≤3 lowered the additive content to below the limit of detection, indicating that all additive had been removed at room temperature within 10 min. This is a significant advancement compared with other solvent‐extraction methods, which need high temperature (reflux) and longer durations (≥1 h). These experiments were then repeated with a second bioinspired additive, diethylenetriamine (DETA), to confirm the robustness of the method. Upon acidification DETA was fully removed from silica in a similar fashion to PEHA‐silica (Figure 1), although with a different pH‐relationship, indicating that acid treatment can be used as a purification method for a wide variety of bioinspired additives.

**Figure 2:**
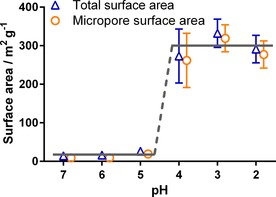
Total and microporous surface area of silica produced using PEHA as measured by *t*‐plot (microporous data offset for clarity), with overlay lines at 30 and 300 m^2^ g^−1^.

**Figure 3:**
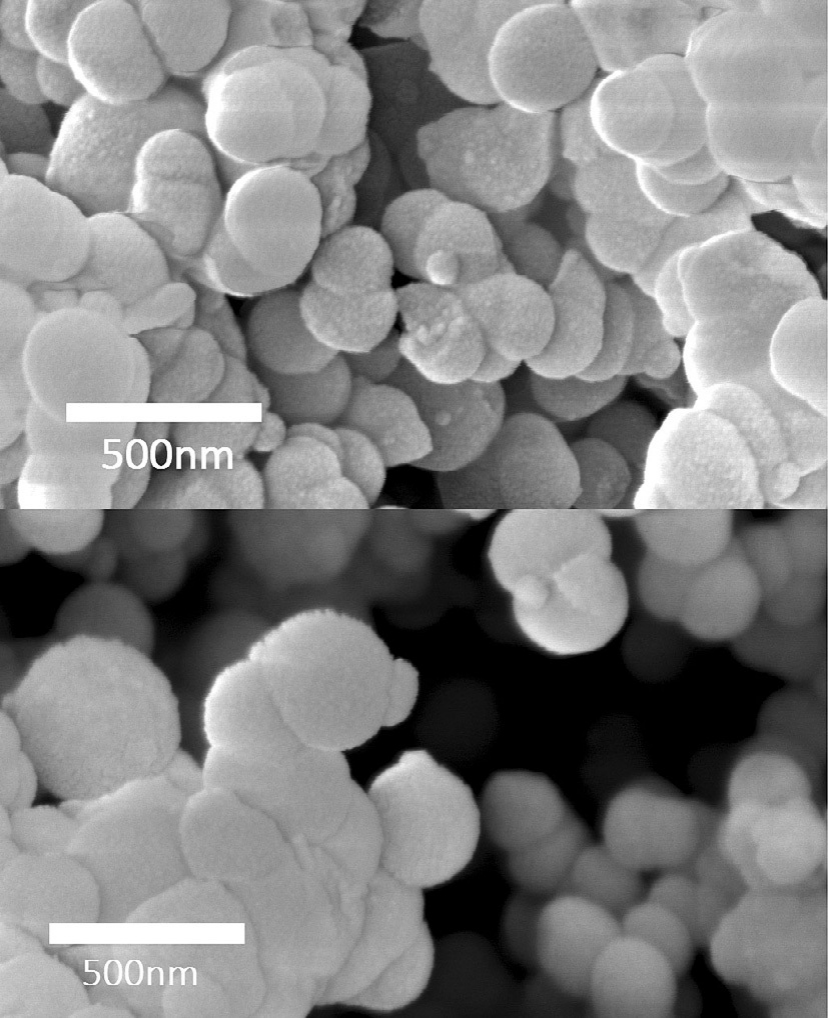
SEM images of PEHA‐silica before (top) and after (bottom) acid treatment, showing no observable change in morphology. Scale bars are 500 nm.

Previous work has shown that purification by calcination introduces porosity to bioinspired silica;^22^ therefore, we measured the effect of acid treatment on the porosity of the samples. Similar to purification with calcination, the surface area increased as the additive was removed (Figure 2) from approximately ≤30 m^2^ g^−1^ at treatment pH≥5 to approximately 300 m^2^ g^−1^ at treatment pH≤4 for both additives. Although the change in porosity occurs less gradually than the changes in composition (Figure 1), it should be noted that the majority of the change occurs between pH 5 and 4 for both composition and porosity (Figure S1 in the Supporting Information). Despite marked changes in porosity, the morphology of the samples remained largely unchanged, as determined by scanning electron microscopy (SEM) (Figure 3).

**Figure 4:**
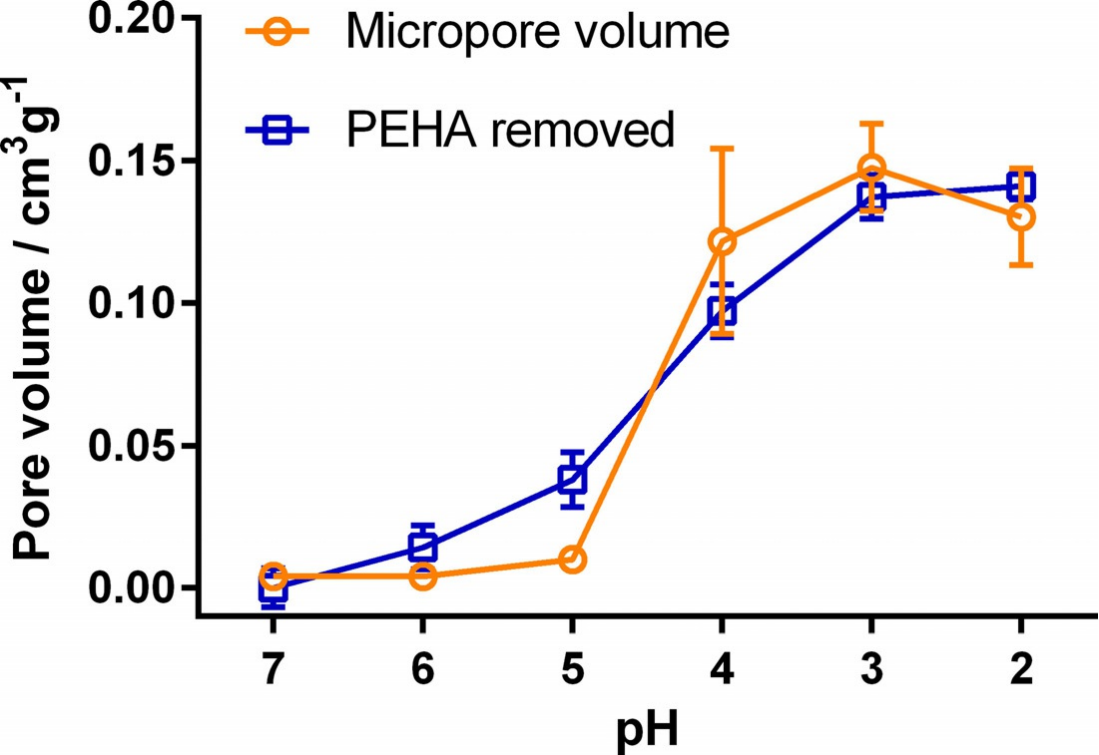
Calculated additive volume removed (squares) and measured micropore volume created from additive removal (circles) against treatment pH value.

The total non‐microporous surface area was found to be <15 m^2^ g^−1^ for all samples using the *t*‐plot method (Figure 2), indicating that all pores generated upon acid treatment were in the microporous region (<2 nm, see Figure S2). Given the size of the PEHA molecules used (reported as 1.8 nm),^24^ it is tempting to assume that the width of each pore created corresponds to the size of an individual PEHA molecule removed. To further support this mechanism, we converted the amount of PEHA removed to the corresponding volume freed and compared it to the volume of micropores created (Figure 4). A good agreement between the measured pore volume of the samples and the estimated volume of additive lost is apparent. The sole exception to this is at pH 5, for which the measured pore volume is lower than the amount of amine removed, which may suggest that the initial additive removed was from the external surface of the material, as has been reported previously.^25^ We will return to this point later.

**Figure 5:**
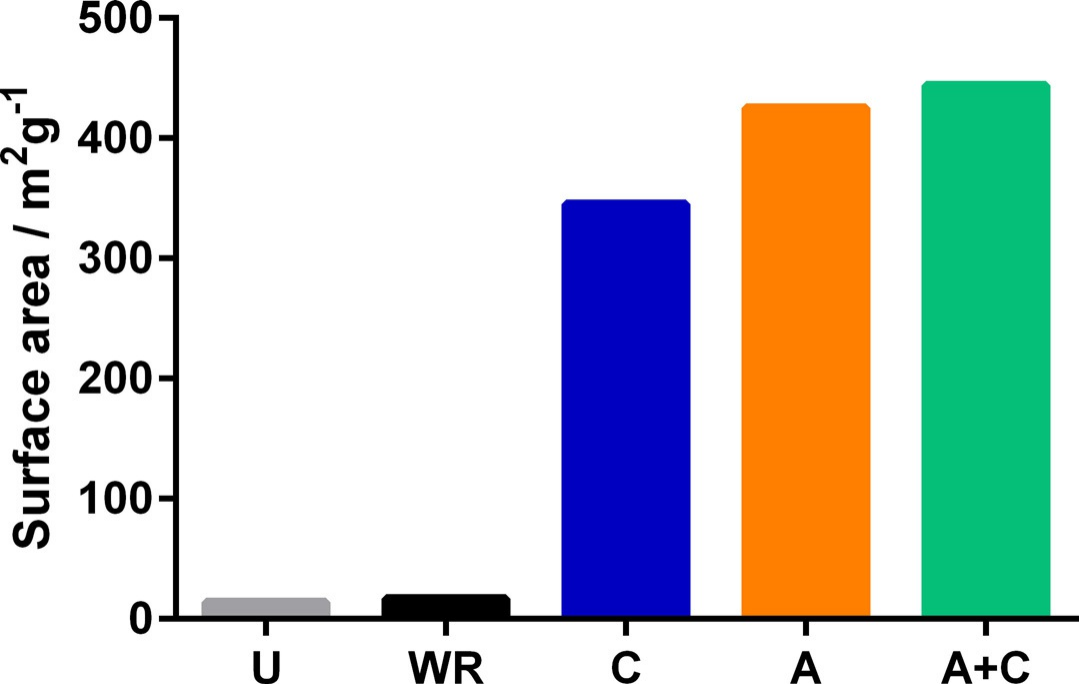
Effect of treatment method on surface area: U=untreated; WR=water reflux; C=calcined; A=acid treated; A+C=acid treated then calcined.

The effectiveness of acid treatment as a purification method was compared against established methods such as high‐temperature solvent extraction and calcination.^4^, ^7^ In particular, the untreated (U) samples were treated under boiling water reflux to emulate ethanol reflux, which has been investigated for templated porous silicas (WR); by calcination (C); or by calcination after acid treatment to pH 2 (A+C), and these treatment methods were compared to acid treatment (A). It was clear that additive removal using water reflux was ineffective (Figure 5). This observation indicated that the driving force of low pH value must be present for additive removal, and also that the U silica composites were highly stable. Calcination increased the surface area of silica similar to the acid treatment (Figure 5); however, the pore‐size analysis showed that larger pores were generated by calcination compared to A or even A+C silica (Figure S2). This degradation of the pore structure, which was only evident for calcined and not for A or A+C silica, indicates that the thermal decomposition of PEHA during calcination causes degradation of the pore structure, an issue that has been reported for calcination of other templated materials.^16^ Therefore, it can be concluded that room‐temperature acid treatment is as effective as calcination in purification, in addition to avoiding the degradation of delicate structures during purification owing to the mild nature of this method compared to conventional techniques. For example, Cassiers et al.^19^ reported complete solvent extraction of template from HMS using acidified water under refluxing conditions whereas Tanev and Pinnavaia^18^ reported the same using neat ethanol. Both methods require high energy inputs and take significantly longer than our method (see energy calculations below). For cetyltrimethylammonium bromide (CTAB)‐templated materials, Hitz and Prins^26^ reported template extraction in ethanol under reflux with 0.1 m salt for 1 h, but their maximum removal efficiency was 73 %, agreeing with the results of Tanev and Pinnavaia.^18^ This contrasts with our results, which provide 100 % removal efficiency in water at room temperature with 0.05 m acid in 10 min. We consider this to be a significant advancement in template removal compared to the literature reports.

### The effect of acid treatment on additive–surface interactions

The need for a pH driving force for the extraction of additive rather than solvothermal conditions indicates a dynamic change in the additive–silica interactions. Understanding these interactions and how they depend on pH value would allow for generalisation of acid treatment to systems other than bioinspired silica, leading to milder purification for a variety of templated materials.

**Figure 6:**
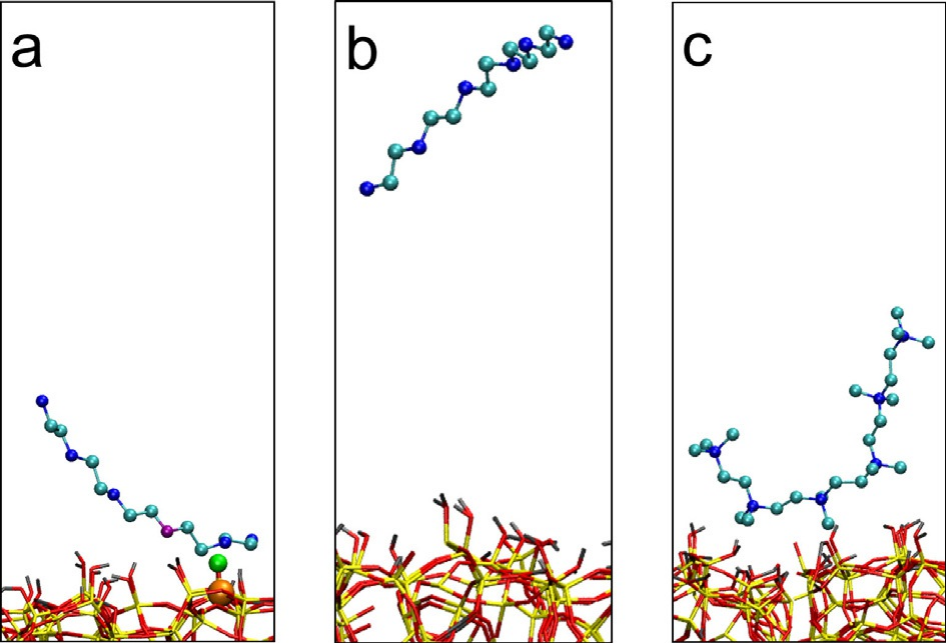
MD simulations depicting (a) interaction between one PEHA amine group and a surface siloxide ion at pH≈7, (b) PEHA desorption after surface neutralisation at pH≈2 and (c) continued adsorption of methyl‐substituted PEHA to silica at pH 2 owing to strong hydrophobic interactions.

The region between pH 4–5 (Figures 1 and 2) is of clear importance because most of the additive removal occurred in that pH range. In this section, we discuss further the mechanism of the removal process to understand the key features of additive removal. Surface–additive interactions in a range of pH 2–7 were simulated using experimentally measured silica‐surface charges^27^ and predicted additive ionisation,^28^ both using the appropriate p*K*a values (see Tables S1 and S8). The results suggest that there are two key interactions controlling the removal of amine additives from silica: ionic attractions between oppositely charged species and solvation of additive in surrounding aqueous solution. However, the balance between these two types of interactions varies with pH value, leading to interesting effects. For example, at pH≥5, the silica surface is negatively and the amine additive positively charged, leading to strong charge–charge interactions between the additive and surface siloxide groups (≡Si−O^−^), consequently resulting in very little to no removal of the additive (Figure 6 a).

Under these conditions, the average separation between surface siloxide anions (<2.6 nm) was significantly larger than the length of the additive (≈1.8 nm) (Table 1). From the simulations, it was observed that each additive molecule can interact with at the most a single siloxide group when adsorbed, regardless of starting position (Figures S5 and S6), meaning that the interaction energies of individual adsorbed molecules over this pH range did not change with pH value (remaining around −250 kJ mol^−1^). Conversely, at pH<4, although additives remain positively charged, the silica surface became predominantly uncharged (Tables 1 and S1^29^). In this case the additive appears to be fully solvated away from the surface (Figure 6 b) because under these conditions the solvation is more energetically favourable than surface–additive interactions (which had been reduced to effectively zero). This explains the complete removal of PEHA under these conditions.

**Table 1:** Surface density of charge as a function of pH value (electrolyte concentration is 0.1 mol dm^−3^ NaCl ionic).^27^

pH	Siloxide ion density^[a]^ [SiO^−^ nm^−2^]	Average siloxide ion separation distance [nm]
3	0	N/A
5^[b]^	0.03	6
7	0.15	2.6

[a] 10 μC cm^−2^=0.6 SiO^−^ nm^−2^ (taken from Ref. 29). [b] Data interpolated.

**Figure 7:**
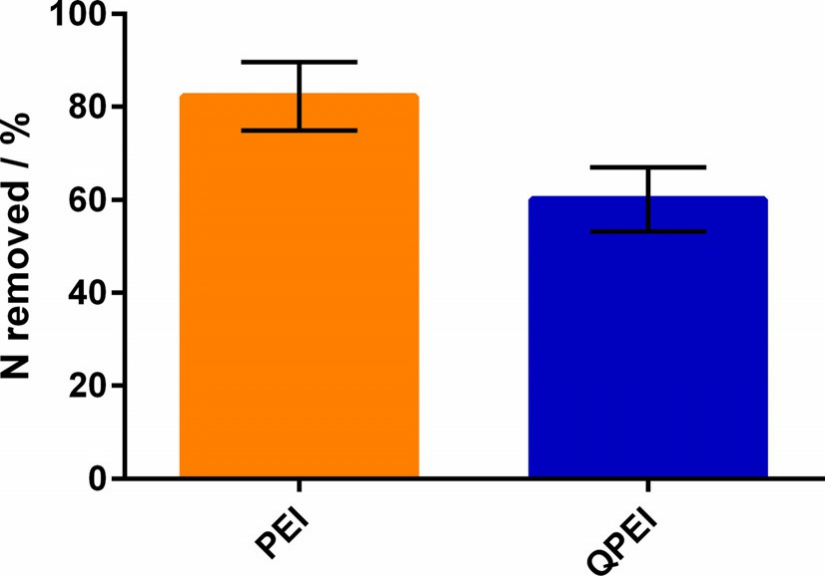
Comparison of additive removal effectiveness between poly(ethyleneimine) (PEI) and methyl‐substituted poly(ethyleneimine) (QPEI), showing 25 % decrease in effectiveness when using QPEI.

To further probe this balance between ionic interactions and additive solvation, we investigated a fully methylated analogue of PEHA (i.e., making their charge permanent, but increasing their hydrophobicity). The results from simulation of the methylated additive indicated that even when no charged interactions were found, solvent extraction became ineffective at pH 2 (Figure 6 c) owing to reduced solvation of the additive and the formation of sufficient non‐ionic interactions with the silica surface (calculated interaction strength of ca. −135 kJ mol^−1^ for methylated PEHA compared to ca. 0 kJ mol^−1^ for PEHA). Experimental data show a 25 % reduction in the removal efficiency for methylated additives compared to their non‐methylated counterparts (Figure 7). Similarly, it has been reported for traditional mesoporous silicas that solvent extraction cannot fully remove methylated templates such as CTAB, whereas non‐methylated counterparts (e.g., DDA) can be completely removed under reflux for 1 h.^18^, ^19^ The removal of CTAB is 25 % less effective than the removal of DDA,^18^ which matches our results and strongly supports our mechanism. The general agreement between simulated and experimental results indicates that MD simulations can be a useful predictive tool for designing non‐destructive template‐removal techniques for templated materials.

Using the simulated interaction strengths, a relationship between the pH value and average surface interaction strength per additive molecule can be inferred [Eq. (1)], in which [≡SiO^−^] and [Additive] represent the number of siloxide ions and additive molecules per area of silica surface, respectively.

(1) $$\left\langle E_{\mathrm{Int}}\right\rangle =\frac{\left[\equiv {\mathrm{SiO}}^{-}\right]}{\left[\mathrm{Additive}\right]}E_{\mathrm{amine}-{\mathrm{SiO}}^{-}}$$

**Figure 8:**
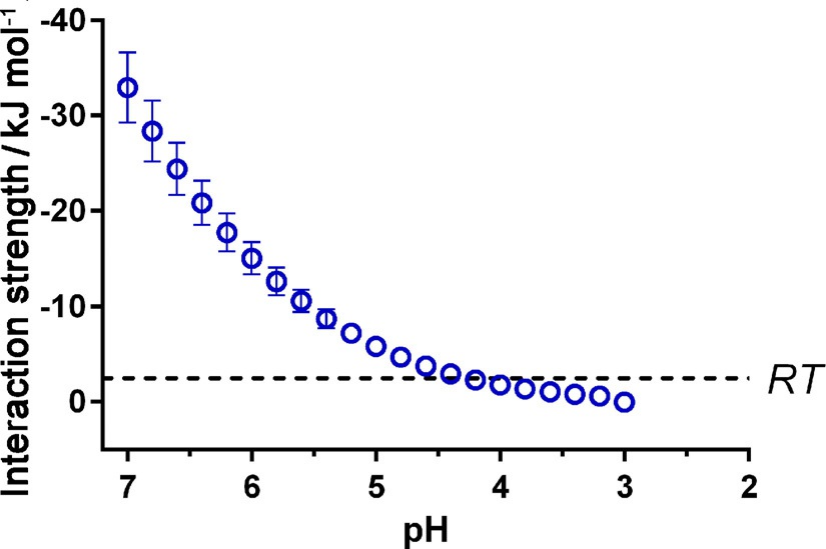
Simulated PEHA–siloxide ion interaction strengths, normalised against availability of precursor ions.

As noted above, in the pH range considered here (pH 2–7), each PEHA molecule can only interact with a single siloxide group and PEHA will always be protonated (partially or fully), hence Equation (1) uses [Additive] for simplicity rather than the concentration of protonated amine moieties. Because [≡SiO^−^] is a function of pH value, the relationship predicted using this equation shows an exponential decrease in the magnitude of interaction strength with reducing pH value (Figure 8), eventually becoming lower than the energy of thermal fluctuations (i.e., *RT*, in which *R* is the ideal gas constant) at approximately pH 4.2. This suggests that above pH 4.2, most additives are still able to (on average) remain attached to the surface, whereas below pH 4.2 thermal fluctuations are sufficient to cause a widespread release of additive molecules, matching our earlier experimental findings for both composition and porosity (Figures 1 and 2).

The simulation results from Figure 8 also imply that all additive should be removed between pH 4–5; however, the experimental results (Figure 1) clearly show that complete removal only occurs at pH<4. Further, the simulation does not explain the discrepancy seen between the amount of the additive removed and the resultant silica pore volume created at pH 5 (Figure 4). To explain these results, the confinement of additives within the silica pore system must be considered.

**Figure 9:**
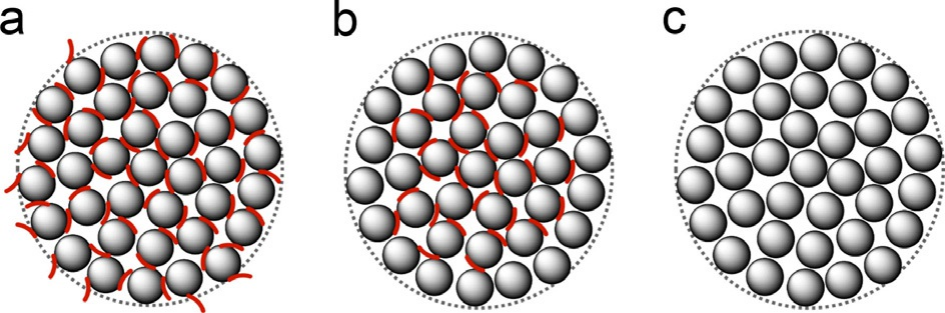
Proposed distribution of additive molecules (red lines) within secondary particles of bioinspired silica (dotted lines), which are composed of primary particles (grey circles). (a) Untreated silica composites contain additives adsorbed to both the particle surface and inside. (b) After partial treatment to pH 5, only the surface‐adsorbed additive molecules are released. (c) After treatment at pH 2, all additive molecules are released.

In the simulations, the additives interact with a flat silica surface, whereas bioinspired silica particles are made up of a network of primary particles (5–10 nm) fused together to form larger secondary particles (>100 nm).^30^ This is depicted in Figure 9, which shows an interstitial pore network in which the majority of additive molecules are likely to be trapped (Figure 9 a). Owing to confinement effects between multiple silica surfaces, the silica–additive interactions for these internally‐held additives are expected to be stronger than at the secondary particle surface (which is geometrically similar to the MD simulations and therefore acts as predicted).^31^ If the pH value is lowered slightly so the interactions are reduced but not eliminated (i.e., pH 5), it is likely that only surface‐bound additives are initially removed owing to their relatively lower interaction strengths (Figure 9 b). Further treatment to lower pH value removes the interaction entirely so that even entrapped additives can be released from the pore structure (Figure 9 c), leaving behind the pure silica network.

This mechanism for initial additive removal from the surface, and subsequently from the interstices, explains the experimental results (Figures 1 and 2) and also the difference between composition and porosimetry data (Figure 4): the removal of surface additive at pH 5 leaves the internal pore structure blocked; therefore, silica surface area and pore volume are largely unaffected. Further treatment to extract internally‐bound additives opens the internal pores to N_2_ adsorption, leading to the sharp change seen in Figure 2 and the convergence of pore volume and additive extracted seen in Figure 4.

### The effect of additive elution on surface chemistry

To experimentally probe the additive localisation, we exploited the specific interaction between CO_2_ and amines through CO_2_ adsorption; in particular, we measured adsorption kinetics and capacity against treatment pH value to assess additive accessibility and functionality, respectively. Because this is a common method to investigate functional porous materials (e.g., proposed carbon‐capture agents),^32^ we further expected CO_2_ sorption to be a good test of whether acid treatment can be used as a single‐step functionalization tool in addition to purification.

**Figure 10:**
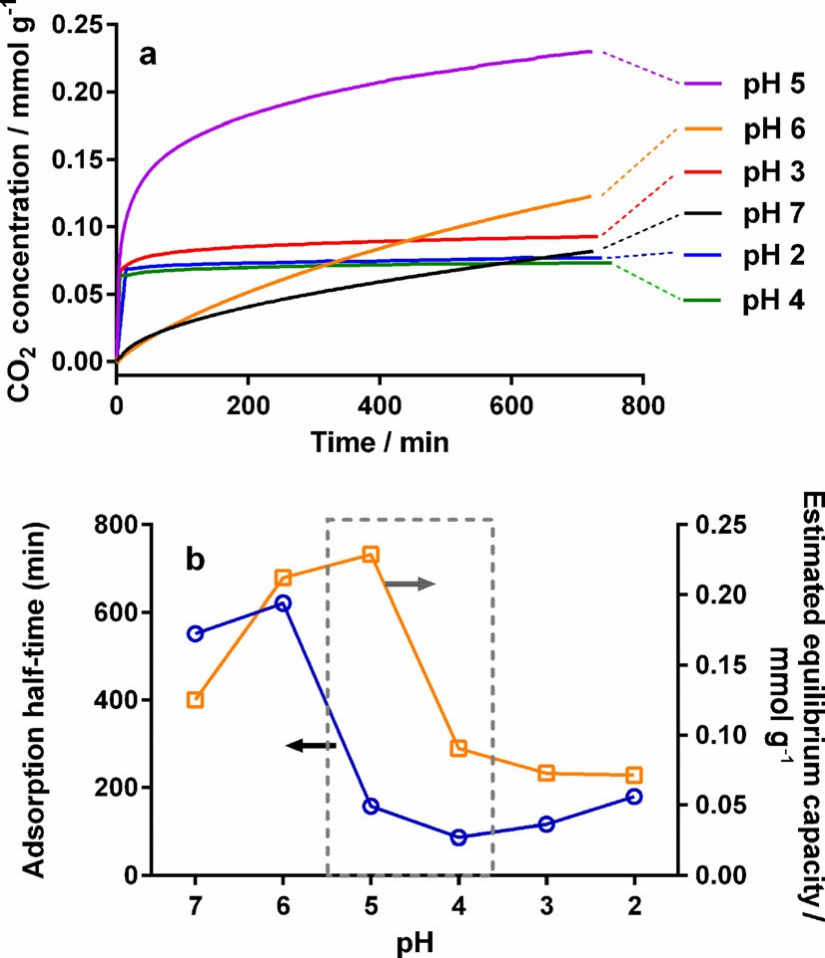
(a) Partial uptake curves at 100 mbar CO_2_ for bioinspired silica treated at different pH values. Data collected prior to pressure stabilisation have been omitted for clarity. (b) Comparison of CO_2_ adsorption half‐times (circles) and equilibrium adsorbed amount (squares) estimated from kinetic‐modelling results.

The measured CO_2_ adsorption rates of different samples at low pressure (Figure 10 a) were analysed to estimate initial adsorption rates (presented as half‐time) and equilibrium capacities (i.e., amount adsorbed) (Figure 10 b). In samples with high additive content (pH≥6), adsorption kinetics were very slow (large half‐times) but the equilibrium capacity was high. This, as well as the low porosity measured using N_2_ adsorption (Figure 2), indicates poor accessibility towards the additives but high chemical activity resulting from the high amine content. This suggests additive surface coverage of the particles (shown in Figure 9 a), which provides a strong driving force for CO_2_ adsorption but blocks access to the pore network, hence slowing the adsorption rate. Conversely, silica with insignificant amounts of amine remaining (pH≤4) showed much faster kinetics arising from the higher surface area, but equilibrium capacities were much lower despite the increase in porosity owing to the absence of amines. This behaviour indicates a change from chemisorption to faster but weaker physisorption brought about by the removal of the amine functionality,^33^ which allows full access to the silica pore system but eliminates the strong driving force for adsorption (Figure 9 c).^34^

Of particular interest was the intermediate case (pH 5), for which the internally bound additives remain within the samples owing to acid treatment. In this case the estimated adsorption capacity was slightly increased compared to the untreated sample even after removal of approximately 25 % of the additive material, whereas the adsorption was much faster (i.e., half‐time was significantly reduced) similar to the purified samples (treated at pH≤4). This indicates that the pH 5 sample exhibited a significant increase in additive accessibility despite the low surface area measured by N_2_ adsorption (Figure 1). These data, along with the discrepancy between sample pore volumes and amount of additive removed at pH 5 (Figure 4), further support the removal of only the surface‐bound additives at pH 5: the access of CO_2_ to the amine‐functionalized internal pores of bioinspired silica is improved, leading to selective adsorption.

The CO_2_ sorption data demonstrate the ability of acid treatment to optimize the surface chemistry of bioinspired silica in a single post‐synthetic step; a technology that is widely investigated for use in post‐combustion carbon‐capture technologies,^35^ although the materials prepared in this work are not intended for use as carbon‐capture sorbents. Acid treatment therefore represents a more environmentally friendly method of not only purifying but also functionalising silica nanoparticles.

### Application of acid‐treatment methods to sustainable engineering

As stated earlier, the drive for developing non‐destructive template‐removal methods has stemmed from the uneconomical and environmentally unfriendly nature of calcination‐based purification. Although this has led to the development of various methods discussed earlier and critically reviewed elsewhere,^4^ we posit that previous approaches did not bring significant enough improvements over calcination to render the production of templated silica materials industrially viable. To this end, in this section we will attempt to demonstrate the industrial viability of bioinspired silica synthesis paired with acid treatment; furthermore, we will estimate and compare the environmental and economic costs of acid treatment, calcination and solvent extraction as described by Tanev and Pinnavaia.^18^

During the course of this study, the bioinspired silica synthesis and acid treatment were successfully performed on a range of scales (150 mL, 500 mL, 1 L and 5 L batch, and 500 mL min^−1^ on a continuous basis). Under continuous operation, we were able to produce up to 300 g of bioinspired silica, demonstrating the scalability of the bioinspired synthesis. The scalability of the acid treatment was tested using a 5 L reactor in batch mode and it was revealed that complete removal of the additive was achieved, similar to the small‐scale results. These experimental results, which are consistent with previous techno‐economic analysis,^13^ clearly demonstrate the scalability of this method and its potential for industrial manufacturing.

When comparing the economic and environmental costs of different purification methods, it is clear from the large variations in operating temperature for each method that matter efficiency alone (e.g., E‐factor) cannot fully describe the relative costs of each purification method. Instead we will estimate energetic costs of each purification method on an industrial scale. In the absence of existing examples of templated‐silica‐process plants, for this study we used our previous techno‐economic analysis of the bioinspired synthesis process.^13^ It was estimated that a calcination furnace for such a plant would require 2.8 MW power or approximately 2000 MJ ton^−1^ silica produced. It was further estimated that a stirred kettle boiler such as would be required for the ethanol reflux used in Ref. 18 would require 5.8 MW power or approximately 4320 MJ ton^−1^ silica. Finally, a stirred tank reactor as would be required for the room‐temperature acid elution described in this study would need only 461 kW, or 84 MJ ton^−1^ silica (only 4 and 2 % of calcination and solvent reflux, respectively). Converting the energetic cost of silica purification for each method to the equivalent CO_2_ emissions,^36^ we find that our new method will reduce the carbon footprint of purification by 95–97 %.

Although the estimated duty values have significant uncertainty (as shown in Figure S7), it is clear that the energetic and environmental costs of acid treatment are orders of magnitude lower than the other methods considered here. Furthermore, because the costs associated with conventional purification are largely thermochemistry‐based (i.e., dependent on the latent heat or heat capacities of the materials involved), there is little room for improvement within these by process intensification. Conversely, such economisation is possible for acid treatment because this process is solely mixing‐ (and therefore equipment‐) dependent.

## Conclusions

In this work we demonstrated a mild, rapid and scalable method of controlling organic additive content in bioinspired silica through post‐synthetic acid treatment. By reducing the pH value of an as‐synthesised silica suspension, we were able to reduce or fully remove the organic additive, concurrently increasing the particle porosity and modulating the silica surface chemistry. Through a combination of experiments and simulations we gained fundamental insight into the surface molecular interactions, enabling us to predict additive‐extraction efficiency. Extraction was found to mainly depend on two sets of pH‐dependent interactions (ionic binding between additive and surface, and solvation energy of additive into solution) although confinement effects also play a role. We could therefore extend the simulations to predict different additive chemistry: as additive hydrophobicity increased, so extraction efficiency decreased. This was found to be qualitatively similar to findings from previous extraction studies for different templated silica materials.

Finally, we demonstrated the economic and environmental viability of our method by estimating both energy requirements and carbon emissions associated with our method and previous, heating‐dependent purification methods. Our method was found to produce orders of magnitude lower carbon emissions, notably being the only method considered that produced fewer emissions during purification than during synthesis. Furthermore, with the ability to directly modify silica surface chemistry rather than using separate purification and functionalisation steps, we were able to entirely eliminate a synthesis step from the production of silica. Therefore, by applying green chemistry principles to the synthesis and purification of bioinspired silica we have developed a cleaner, cheaper and readily scalable method for silica production, a significant technological advance.

## Experimental Section

### Synthesis

Bioinspired silica was synthesised at 150 mL scale by mixing solutions of sodium silicate pentahydrate (Fisher scientific, technical grade) and pentaethylenehexamine (Sigma–Aldrich, technical grade) in deionised water so that the final concentrations were 30 mm for both Si and N. The mixture was subsequently neutralised using 1 m HCl and allowed to react at pH 7.0±0.05 for 5 min. Particles were isolated by centrifugation for 15 min at 4500 g and washing with deionised water, repeated three times, before being dried in an oven at 85 °C overnight. When performing the reaction on a large scale, a 1 or 5 L Reactor‐ReadyTM system was used either in batch or continuous mode. The reactor stirrer was set to 500 rpm, and other parameters and procedures were as described above. Alternative additives used were diethylenetriamine (Sigma–Aldrich, 99 %) at an N concentration of 30 mm, and poly(ethyleneimine) and permethylated poly(etheleneimine) (Polysciences) at a concentration of 1 mg mL^−1^.

### Purification

Acid extraction was performed on silica suspended within the reaction mixture by addition of further HCl to lower the pH to a desired value, and left to stand for approximately 10 min before being isolated by centrifuge as described above. Water reflux was performed by suspending bioinspired silica (ca. 0.75 g) in deionised water (120 mL) and heating to reflux at 100 °C for 24 h. The sample was then allowed to cool to ambient temperature before being filtered and washed with further deionised water (120 mL). The washed sample was then dried at 85 °C overnight prior to subsequent analyses. Calcination was performed in an Elite TSH15 tube furnace in which the sample (ca. 0.5 g) was heated at a ramp rate of 10 °C min^−1^ to 550 °C and held under flowing N_2_ for 8 h. The silica was then cooled to ambient temperature and collected for further analysis.

### Analysis

Elemental (CHN) analysis of the silica was performed on a PerkinElmer 2400 Series II CHNS Analyser. To measure porosity from N_2_ adsorption (Micromeritics ASAP 2420), dried powders were first degassed at 110 °C and 5 μm Hg for 2 h. N_2_ was then dosed onto the sample at 77 K and the volume adsorbed was measured as a function of pressure. This was used to calculate the BET surface area,^37^ Barrett–Joyner–Halenda (BJH) pore‐size distribution^38^ and *t*‐plot microporous surface area^39^ of the samples. To further probe porosity and investigate functionality, silica samples were tested for CO_2_ adsorption in a Hiden Isochema Intelligent Gravimetric Analyser (IGA). Silica samples were loaded onto the IGA, after which they were degassed at high vacuum and 120 °C for 4 h. CO_2_ was then fed into the analysis chamber under constant pressure for 12 h and the CO_2_ adsorbed was measured. A double‐exponential model, which has been reported as a catch‐all numerical model for describing adsorption processes,^40^ was used to model the kinetics of adsorption.

### Simulations

Molecular dynamics simulations were performed using the GROMACS software^41^ to study the interactions between amine molecules and silica surfaces at different pH values. A slab of amorphous silica with a thickness of approximately 2.5 nm and a cross‐sectional area of 18.3 nm^2^ was built from structures provided by Heinz et al.,^42^ in which the silica surface has a density of silanol groups of 4.7 nm^−2^ and a degree of ionization dependent on solution pH (Table S1). A pre‐equilibrated slab of water containing a single amine molecule (i.e., simulating the infinite dilution limit) was placed above the silica slab and three‐dimensional periodic boundary conditions were applied (Figure S5 and Tables S2–S6). Simulations were run in the canonical (NVT) ensemble at 298 K using standard parameters.

To confirm equilibrium had been reached in each case, two different starting configurations were tested until simulated energies were independent of the starting positions: one in which the amine was placed in the centre of the water slab and another in which the amine was placed as close to the surface as possible (see Figures S5 and S6), following an energy optimization in vacuum. To discount the possibility of finite‐size effects on the simulated energies, box lengths of 7 and 14 nm perpendicular to the surface were both tested.

PEHA molecules were modelled using the OPLS‐AA potential,^43^ water by the SPC/E model^44^ and silica by the INTERFACE potential, which has been used successfully to study adsorption of amine‐containing‐peptides onto silica surfaces.^42^, ^45^, ^46^ The latter was validated for use within the OPLS framework by calculating the heat of immersion of the silica surface following the procedure described by Emami et al.^45^ (see the Supporting Information for details). To estimate the interaction energy between amines and the silica surface at different pH values, we simulated surfaces with degrees of ionization corresponding to experimental titration measurements of amorphous silica^27^ −0 % for pH<3.5, 0.6 % for pH 5 (corresponding to a single ≡SiO^−^ group in the simulation box) and 2.4 % for pH 6.5 (Table S1). The degree of protonation of the amines as a function of pH value was determined from the SPARC online calculator (Table S8).^28^ Charge neutrality was ensured by adding sodium and chloride ions to the simulation box as needed. Input files for all simulations performed in this work are openly available from the University of Strathclyde data repository at 10.15129/baf10272‐6834‐4cad‐8b1a‐7c73f406d0ce.

## Conflict of interest

*The authors declare no conflict of interest*.

## Supporting information

As a service to our authors and readers, this journal provides supporting information supplied by the authors. Such materials are peer reviewed and may be re‐organized for online delivery, but are not copy‐edited or typeset. Technical support issues arising from supporting information (other than missing files) should be addressed to the authors.

Supplementary
