# Supplementary material for: An Eco‐Friendly, Tunable and Scalable Method for Producing Porous Functional Nanomaterials Designed Using Molecular Interactions
Source: ChemSusChem. 2017 Mar 29;10(8):1683–91. doi: 10.1002/cssc.201700027 (PMC5434938; doi:10.1002/cssc.201700027)
Supplement: Supplementary file 1 — Supplementary [file CSSC-10-1683-s001.pdf]

CHEMISTRY & SUSTAINABILITY
CHEMSUSCHEM
ENERGY & MATERIALS

Supporting Information

An Eco-Friendly, Tunable and Scalable Method for Producing Porous Functional Nanomaterials Designed Using Molecular Interactions

Joseph R. H. Manning,[a] Thomas W. S. Yip,[b] Alessia Centi,[b] Miguel Jorge,[b] and Siddharth V. Patwardhan*[a]

cssc_201700027_sm_miscellaneous_information.pdf

Change in PEHA concentration after acid treatment to different pH

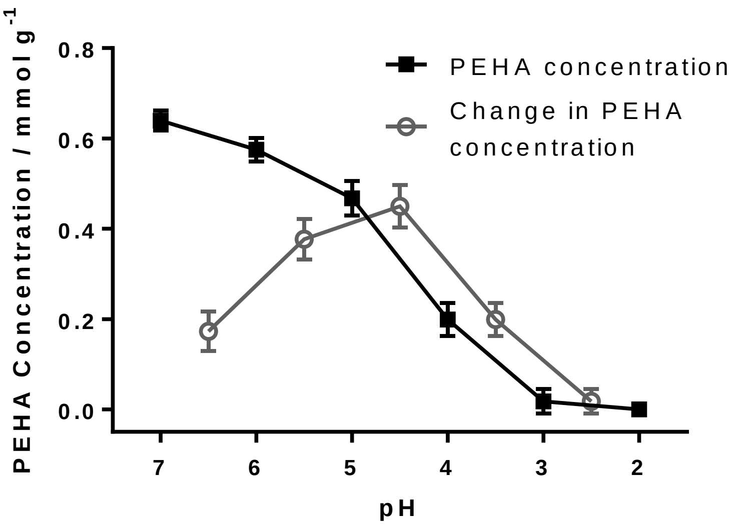

Figure S1. Graph of additive content in silica versus acidification pH (squares). Circles show change in PEHA concentration between different acid treatment levels (i.e. circle at pH 4.5 shows the difference in concentration between pH 5 and pH 4)

Pore-size analysis of bioinspired silica after additive removal:

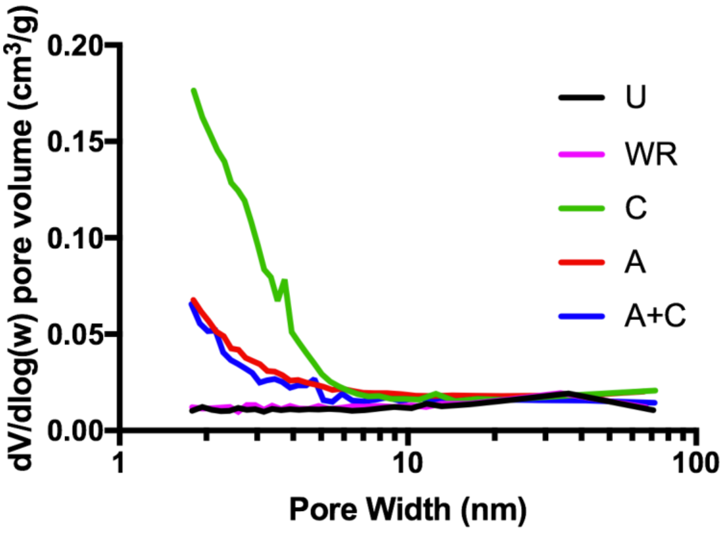

Figure S2. Effect of treatment method on desorption pore-size. Methods as follows: U – no treatment; WR – Water Reflux; C – calcined; A – Acid treated; A+C – acid treated then calcined.

Surface-charge calculations on bioinspired silica:

Table S1. Surface density of charge as a function of pH at electrolyte concentration 0.1 mol/dm^3 NaCl ionic, σ in μCcm^{-3}. [1]

pH	Surface charge density (μC/cm^2)	Siloxide ion density (SIO$^-$/nm^2) [a)]	Number of siloxide ions in simulation box	Average siloxide ion separation distance (nm)	% surface charge
3	0	0	0	0	0
3.5[b)]	0.08	0.005	0.18	14.7	0.10
4[b)]	0.15	0.01	0.34	10.5	0.19
4.5[b)]	0.27	0.02	0.60	7.8	0.35
5[b)]	0.46	0.03	1	6	0.59
5.5[b)]	0.74	0.04	1.62	4.7	0.94
6[b)]	1.14	0.07	2.5	3.8	1.46
6.5	1.86	0.11	4.08	3.0	2.37
7	2.48	0.15	5.44	2.6	3.17
7.5	3.38	0.20	7.42	2.2	4.31
8	4.31	0.26	9.46	2.0	5.50
8.5	5.86	0.35	12.86	1.7	7.48
9	8.34	0.50	18.32	1.4	10.65
9.5	13.27	0.80	29.14	1.1	16.94

10 μC/cm^2=0.6 SiO$^-$/nm^2; [b)] Data interpolated;

Molecular dynamics Model details and validation: calculation of the heat of immersion

All amine species in our system were modelled using the OPLS all-atom force field[2] (see parameters in Tables S2-S5 and Figure S2) combined with the SPC/E model for water[3]. Parameters for silica surfaces were taken from the INTERFACE force field[4]. This is ready to use in combinations with several other force fields such as AMBER, CHARMM, CVFF, PCFF, COMPASS, however some adjustments were necessary to use it in the OPLS framework due to the different conventions adopted in the OPLS and in CHARMM force fields for dealing with the 1-4 interactions (CHARMM takes 100% of the 1-4 interactions whereas OPLS only takes 50% of them). To account for this difference, additional terms for Lennard-Jones interactions were added in the silica surface parameters (see Table S6).

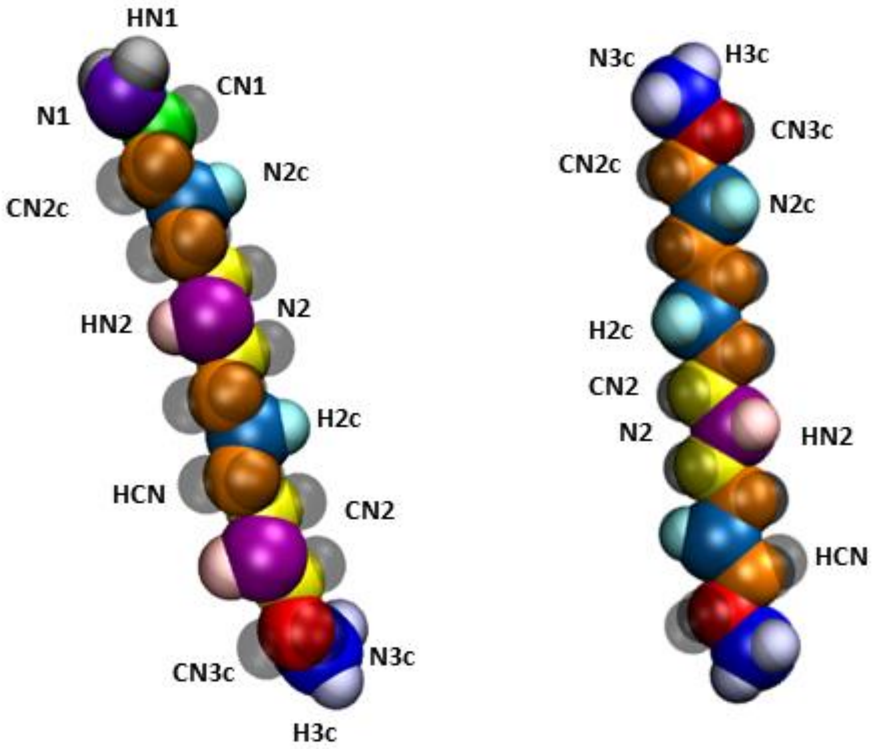

Figure S3. Schematic representation of two amine topologies covering all different atom types: N1 - violet, N2 - purple, N3c - blue, N2c - light blue, HN1 - gray, HN2 - pink, H3c - ice blue, H2c - cyan, HCN - black, CN1 - green, CN2 - yellow, CN2c - orange, CN3 - red.

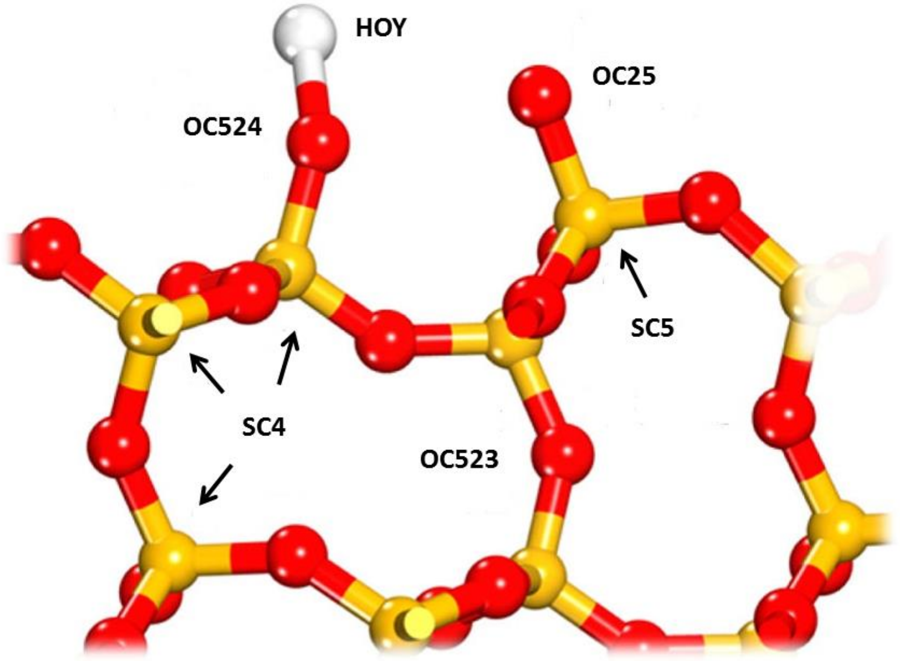

Figure S4. Schematic representation of the silica model showing the different atom types: Hydrogen – white, Silicon – yellow, Oxygen - red. Figure adapted from Emami[5].

Table S2. Lennard-Jones parameters, point charges and atomic masses for all atom types. Values for silica surfaces have been adapted from Emami[5] to be used in GROMACS 4.6 software[6] in the OPLS framework.

Site	Mass (a.u.)	q (C)	σ (nm)	ε (kJ mol^{-1})
**SC4**	28.0860	1.1	0.369724	0.389112
**SC5**	28.0860	0.725	0.369724	0.389112
**OC23**	15.9994	-0.55	0.30914	0.225936
**OC24**	15.9994	-0.675	0.30914	0.510448
**OC25**	15.9994	-0.9	0.30914	0.510448
**HOY**	1.0080	0.4	0.09667	0.06276
**NA+**	22.98977	1	0.282415	0.393296
**N1**	14.0067	-0.9	0.33	0.71128
**N2**	14.0067	-0.78	0.33	0.71128
**CN1**	12.011	0.06	0.35	0.276144
**CN2**	12.011	0.08	0.35	0.276144
**HN1**	1.008	0.36	0	0
**HN2**	1.008	0.38	0	0
**HCN**	1.008	0.06	0.25	0.06576
**N3c**	14.0067	-0.3	0.325	0.71128
**H3c**	1.008	0.33	0	0
**CN3c**	12.011	0.19	0.35	0.276144
**N2c**	14.0067	-0.2	0.325	0.71128
**H2c**	1.008	0.31	0	0
**CN2c**	12.011	0.17	0.35	0.276144

Table S3. Bond lengths, and harmonic force constants for silica surfaces and amines.

**Bond**	**b_0 (nm)**	**k_b (kJ mol^{-1}nm^{-2})**
**SC4-OC23**	0.165	238488
**SC4-OC24**	0.165	238488
**SC5-OC23**	0.165	238488
**SC5-OC24**	0.165	238488
**SC5-OC25**	0.165	238488
**OC24-HOY**	0.1471	414216
**N1-HN1**	0.101	363171.2
**N2-HN2**	0.101	363171.2
**N3c-H3c**	0.101	363171.2
**N2c-H2c**	0.101	363171.2
**N1-CN1**	0.1448	319657
**N2-CN2**	0.1448	319657
**N3c-CN3c**	0.1471	307105.6
**N2c-CN2c**	0.1471	307105.6
**CN2-CN2**	0.1529	224262.4
**CN2-CN3c**	0.1529	224262.4
**CN1-CN2c**	0.1529	224262.4
**CN2-CN2c**	0.1529	224262.4
**CN2c-CN3c**	0.1529	224262.4
**CN2c-CN2c**	0.1529	224262.4
**CN1-HCN**	0.109	284512
**CN2-HCN**	0.109	284512
**CN3c-HCN**	0.109	284512
**CN2c-HCN**	0.109	284512

Table S4. Bond angles and harmonic force constants for silica surfaces and amines.

**Angles**	θ_0 (deg)	k_θ (kJ mol^{-1}rad^{-2})
**SC4-OC23-SC4**	149.0	836.8
**SC4-OC24-HOY**	115.0	418.4
**SC5-OC23-SC4**	149.0	836.8
**SC5-OC24-HOY**	115.0	418.4
**OC23-SC4-OC23**	109.5	836.8
**OC23-SC4-OC24**	109.5	836.8
**OC23-SC5-OC23**	109.5	836.8
**OC23-SC5-OC24**	109.5	836.8
**OC23-SC5-OC25**	109.5	836.8
**OC24-SC4-OC24**	109.5	836.8
**OC24-SC5-OC24**	109.5	836.8
**HN1-N1-HN1**	106.4	364.845
**CN1-N1-HN1**	109.5	292.88
**CN2-N2-HN2**	109.5	292.88
**HCN-CN1-N1**	109.5	292.88
**HCN-CN2-N2**	109.5	292.88
**H3c-N3c-H3c**	109.5	292.88
**H2c-N2c-H2c**	109.5	292.88
**CN3c-N3c-H3c**	109.5	292.88
**CN2c-N2c-H2c**	109.5	292.88
**HCN-CN3c-N3c**	109.5	292.88
**HCN-CN2c-N2c**	109.5	292.88
**CN2-CN2-N2**	109.47	470.281
**N1-CN1-CN2c**	109.47	470.281
**N2-CN2-CN2c**	109.47	470.281
**N2-CN2-CN3c**	109.47	470.281
**HCN-CN1-HCN**	107.80	276.144
**HCN-CN2-HCN**	107.80	276.144
**HCN-CN3c-HCN**	107.80	276.144
**HCN-CN2c-HCN**	107.80	276.144
**CN2-CN2-HCN**	110.70	313.8
**CN2c-CN3c-HCN**	110.70	313.8
**CN2c-CN1-HCN**	110.70	313.8
**CN3c-CN2c-HCN**	110.70	313.8
**CN3c-CN2-HCN**	110.70	313.8
**CN2-CN3c-HCN**	110.70	313.8
**CN1-CN2c-HCN**	110.70	313.8
**CN2c-CN2c-HCN**	110.70	313.8
**CN2-CN3c-N3c**	111.20	669.44
**CN1-CN2c-N2c**	111.20	669.44
**CN2-CN2c-N2c**	111.20	669.44
**CN3c-CN2c-N2c**	111.20	669.44
**CN2c-CN3c-N3c**	111.20	669.44
**CN2c-CN2c-N2c**	111.20	669.44
**CN2-CN3c-N3c**	111.20	669.44
**CN2c-N2c-CN2c**	113.00	418.4
**CN2-N2-CN2**	107.20	433.462

Table S5. Dihedral torsion parameters for amines.

**Dihedral**	**C_0 (kJ/mol^{-1})**	**C_1 (kJ/mol^{-1})**	**C_2 (kJ/mol^{-1})**	**C_3 (kJ/mol^{-1})**	**C_4 (kJ/mol^{-1})**	**C_5 (kJ/mol^{-1})**
**H3c-N3c-CN3c-HCN**	0.54601	1.63803	0	-2.18405	0	0
**H2c-N2c-CN2c-HCN**	0.54601	1.63803	0	-2.18405	0	0
**H3c-N3c-CN3c-CN2c**	-1.26775	3.02085	1.74473	-3.49782	0	0
**H2c-N2c-CN2c-CN3c**	-1.26775	3.02085	1.74473	-3.49782	0	0
**H2c-N2c-CN2c-CN2c**	-1.26775	3.02085	1.74473	-3.49782	0	0
**HN2-N2-CN2-CN3c**	-1.26775	3.02085	1.74473	-3.49782	0	0
**HN2-N2-CN2-CN2c**	-1.26775	3.02085	1.74473	-3.49782	0	0
**H2c-N2c-CN2c-CN2**	-1.26775	3.02085	1.74473	-3.49782	0	0
**HN1-N1-CN1-CN2c**	-1.26775	3.02085	1.74473	-3.49782	0	0
**H2c-N2c-CN2c-CN1**	-1.26775	3.02085	1.74473	-3.49782	0	0
**N3c-CN3c-CN2c-N2c**	19.59994	-21.3907	4.05011	-2.25936	0	0
**N2c-CN2c-CN2c-N2c**	19.59994	-21.3907	4.05011	-2.25936	0	0
**N3c-CN3c-CN2-N2**	19.59994	-21.3907	4.05011	-2.25936	0	0
**N2-CN2-CN2c-N2c**	19.59994	-21.3907	4.05011	-2.25936	0	0
**N1-CN1-CN2c-N2c**	19.59994	-21.3907	4.05011	-2.25936	0	0
**N2-CN2-CN2-N2**	19.59994	-21.3907	4.05011	-2.25936	0	0
**N3c-CN3c-CN2c-HCN**	0.80333	2.40999	0	-3.21331	0	0
**N2c-CN2c-CN2c-HCN**	0.80333	2.40999	0	-3.21331	0	0
**N3c-CN3c-CN2-HCN**	0.80333	2.40999	0	-3.21331	0	0
**HCN-CN3c-CN2c-HCN**	0.62760	1.88280	0	-2.5104	0	0
**HCN-CN2c-CN2c-HCN**	0.62760	1.88280	0	-2.5104	0	0
**HCN-CN3c-CN2-HCN**	0.62760	1.88280	0	-2.5104	0	0
**HCN-CN1-CN2c-HCN**	0.62760	1.88280	0	-2.5104	0	0
**HCN-CN2-CN2-HCN**	0.62760	1.88280	0	-2.5104	0	0
**HCN-CN3c-CN2c-N2c**	0.97069	2.91206	0	-3.88275	0	0
**HCN-CN2-CN2c-N2c**	0.97069	2.91206	0	-3.88275	0	0
**HCN-CN1-CN2c-N2c**	0.97069	2.91206	0	-3.88275	0	0
**CN3c-CN2c-N2c-CN2c**	3.04176	-1.35144	-0.51881	2.20915	0	0
**CN2c-N2c-CN2c-CN2c**	3.04176	-1.35144	-0.51881	2.20915	0	0
**CN1-CN2c-N2c-CN2c**	3.04176	-1.35144	-0.51881	2.20915	0	0
**HCN-CN2c-N2c-CN2c**	0.63179	1.89535	0	2.52714	0	0
**HCN-CN3c-CN2-N2**	-4.09614	5.08775	2.96645	-3.95806	0	0
**HCN-CN2c-CN1-N1**	-4.09614	5.08775	2.96645	-3.95806	0	0
**CN3c-CN2-N2-CN2**	1.78866	3.49154	0.53555	-5.81576	0	0
**CN2c-CN2-N2-CN2**	1.78866	3.49154	0.53555	-5.81576	0	0
**HCN-CN2-N2-HN2**	0.83680	2.51040	0	-3.34720	0	0
**HCN-CN2-N2-CN2**	1.17152	3.51456	0	-4.68608	0	0

Table S6. Parameters for 1-4 interactions in silica surfaces and amines.

Interaction	C^6 (nm)	C^{12} (kJ mol^{-1})
**SC4-SC4**	0.369724	0.389112
**SC4-SC5**	0.369724	0.389112
**SC4-OC23**	0.338078	0.296504
**SC4-OC24**	0.338078	0.44567
**SC4-OC25**	0.338078	0.44567
**SC4-HOY**	0.189053	0.156271
**SC5-SC5**	0.369724	0.389112
**SC5-OC23**	0.338078	0.296504
**SC5-OC24**	0.338078	0.44567
**SC5-OC25**	0.338078	0.44567
**SC5-HOY**	0.189053	0.156271
**OC23-OC23**	0.309140	0.225936
**OC23-OC24**	0.309140	0.339601
**OC23-OC25**	0.309140	0.339601

To make sure that our approach based on OPLS with the appropriate corrections is equivalent to the CHARMM approach in the original force field, we calculated the heat of immersion of a pyrogenic silica surface with 0% ionization and compared this value with the value reported in reference[4]. The pyrogenic silica surface was used to match the original force field validation protocol. All parameters are exactly the same as those used to describe amorphous silica surfaces in this work. In addition to this, in all our simulations we use the SPC/E water model[3] whereas Emami[5] tested silica-water interfacial properties for SPC, TIP3P and PCFF water models. Hence, calculation of the heat of immersion in our system provides not just an indication of the transferability of the INTERFACE force field within the OPLS framework but also of its performance in combination with a different water model. As such, four different systems were considered: 1) CHARMM framework with SPC water, 2) CHARMM framework with SPC/E water, 3) OPLS framework with additional non-bonded interactions and SPC water and 4) OPLS framework including non-bonded interactions and SPC/E water.

The heat of immersion is defined as:

$$\Delta_{\mathrm{H}} = \frac{E_{surface-in-water} - E_{surface-in-vacuum} - E_{water}}{2A} \qquad (1)$$

Where $E_{\text{surface-in-water}}$ is the total energy of the surface immersed in water, $E_{\text{surface-in-vacuum}}$ the energy of the surface in vacuum and E_{water} the energy of a box containing 1600 water molecules. This energy is normalised by the total surface area (2A) so that units are mJ/m^2

The computational procedure[5] adopted requires that, for each of the systems studied, three molecular simulations corresponding to the three energy terms in the equation (with the exception of the vacuum term which is independent of the model used for water) are performed. Once again, structures and topology of pyrogenic silica were taken from the INTERFACE package and adapted to be used in GROMACS 4.6 software. The system representing the surface immersed in water was created by

adding 1600 water molecules to a simulation box containing the surface. This was followed by energy minimisation and equilibration in the NpT ensemble with semi-isotropic pressure coupling at 273 K before data were collected at 300 K in the NvT ensemble for 6 ns and using a time step of 1 fs. The exact same steps of equilibration were followed also for the system containing only 1600 water molecules, whereas the system in vacuum was created by deleting all the water molecules from the surface-in-water system while extending the box height to 30 nm. In this last case no NpT equilibration is required. A cut-off of 1.2 nm was applied to short-range dispersion interactions and the same distance was used for the particle-mesh Ewald method (PME)[7] to take into account the long-range Coulomb electrostatics. Finally, a long-range dispersion correction term was added to both energy and pressure. For all simulations, total energies were averaged over the last 4 ns providing the results shown in Table S7.

Table S7. Comparison of heat of immersion of pyrogenic silica surface in water

Publication	ΔH (model used) (mJm^{-2})
This work	157 ± 1 (CHARMM-SPC), 199 ± 1 (CHARMM-SPC/E), 157 ± 1 (OPLS-SPC), 167 ± 2 (OPLS-SPC/E)
Emami [4]	167 ± 2 (SPC), 160 ± 2 (TIP3P), 157 ± 2 (PCFF)
Taylor [7]	160 ± 5

Our results show, with the exception of the system in the CHARMM framework using the SPC/E water model, very good agreement with both experimental[8] and previous simulation results[5] confirming that our approach using the OPLS framework and SPC/E model for water can be considered a valid approximation to represent interactions with silica surfaces, including amorphous surfaces which are the main interest of this work.

Effect of starting position on simulation endpoint:

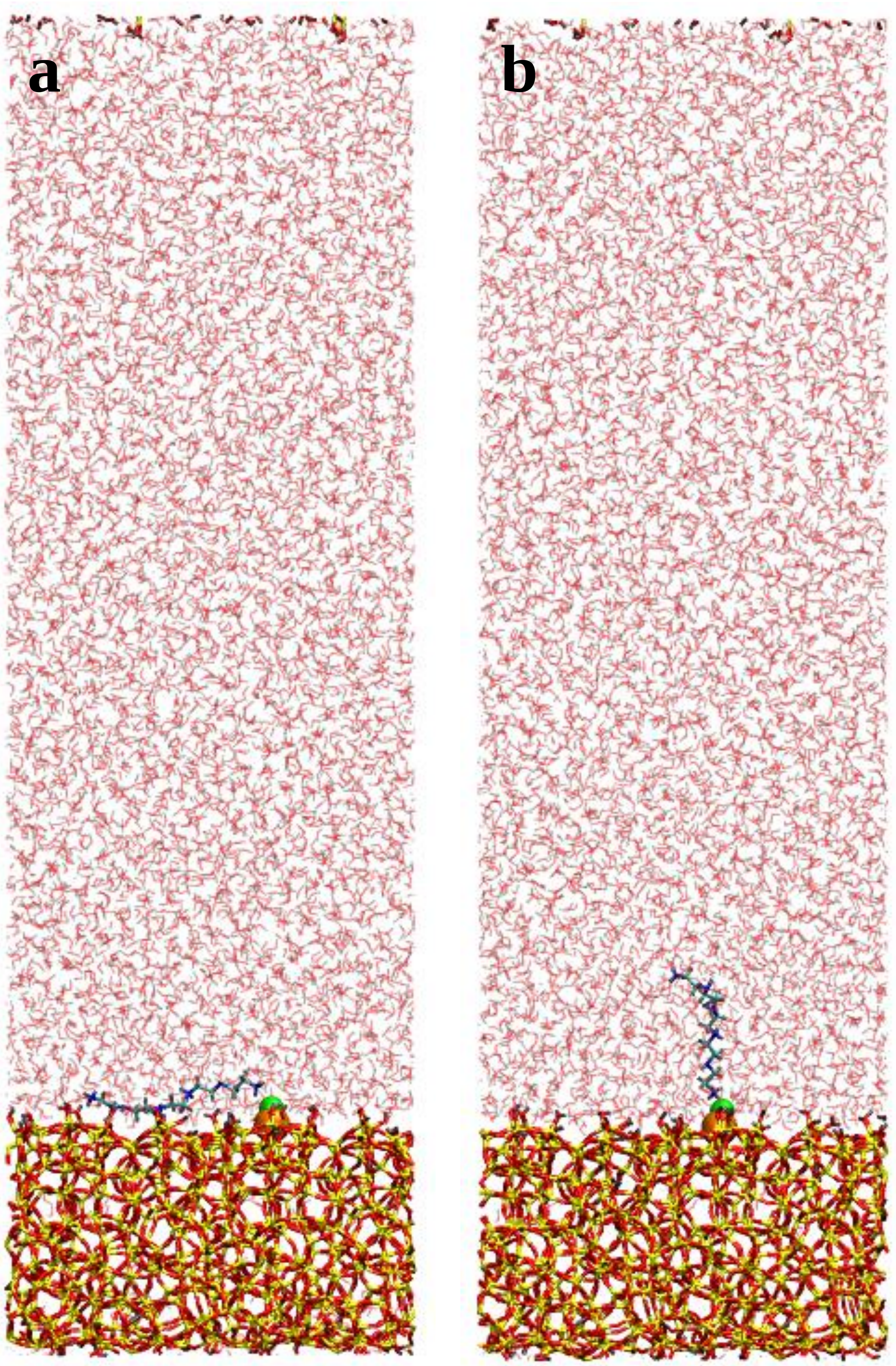

Figure S5. Snapshots representing the initial (a) and final (b) configuration for the system at pH=5 when starting with the amine close to the surface.

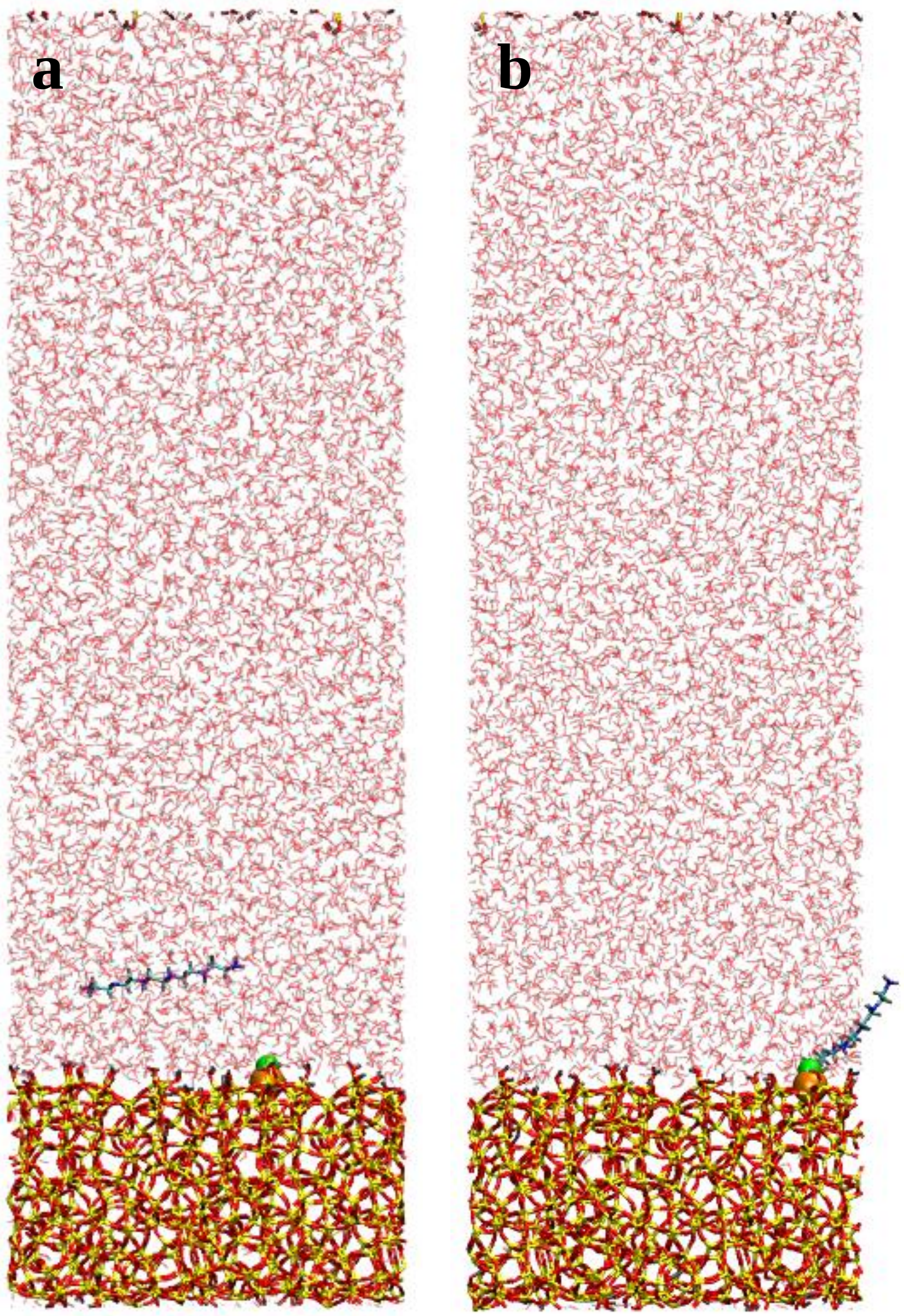

Figure S6. Snapshots representing the initial (a) and final (b) configuration for the system at pH=5 when starting with the amine away from the surface.

Calculated PEHA species populations:

Table S8. Population of amines between pH 3 and 7. Ionisation data obtained from the SPARC online calculator[9], N^0 - unionised, N^+ - ionised.

Species :	$N^+N^+N^+N^+N^+N^+$	$N^+N^0N^+N^+N^+N^+$	$N^+N^+N^0N^+N^+N^+$	$N^+N^0N^+N^0N^+N^+$	$N^+N^0N^+N^+N^0N^+$	$N^0N^+nN^0N^+N^0N^+$	$N^+N^0N^0N^+N^0N^+$
pH							
3	0.51	0.13	0.37	0.01	0.01	0	0
3.2	0.39	0.16	0.45	0.03	0.01	0	0
3.4	0.28	0.19	0.51	0.05	0.02	0	0
3.6	0.19	0.2	0.55	0.08	0.03	0	0
3.8	0.12	0.2	0.55	0.13	0.06	0	0
4	0.07	0.19	0.52	0.2	0.08	0	0
4.2	0.04	0.16	0.45	0.28	0.12	0	0
4.4	0.02	0.14	0.38	0.37	0.15	0	0
4.6	0.01	0.11	0.29	0.45	0.19	0	0
4.8	0	0.08	0.21	0.52	0.22	0	0.01
5	0	0.05	0.15	0.58	0.24	0	0.01
5.2	0	0.04	0.1	0.62	0.26	0	0.02
5.4	0	0.02	0.07	0.64	0.27	0.01	0.03
5.6	0	0.02	0.04	0.65	0.27	0.01	0.05
5.8	0	0.01	0.03	0.64	0.26	0.02	0.07
6	0	0.01	0.02	0.61	0.25	0.03	0.11
6.2	0	0	0.01	0.57	0.24	0.04	0.16
6.4	0	0	0.01	0.51	0.21	0.06	0.23
6.6	0	0	0	0.44	0.18	0.08	0.31
6.8	0	0	0	0.35	0.15	0.11	0.4
7	0	0	0	0.27	0.11	0.13	0.48

Details of calculations of energy requirements for calcination and acid treatment

Calcination

On the lab scale, the energy required for calcination depends on the equipment power rating only, and does not vary with the amount of material being treated at any time. Therefore, it is impossible to extrapolate process-scale-relevant information from small-scale experiments. One important observation, however, is that the relative power requirements for heating and maintaining temperature are approximately 75% and 20% of the total equipment power rating, respectively.

Unlike lab-scale furnaces industrial furnaces can achieve a fuel efficiency of approximately 80%[10], which allows for design based on material throughput. Heat capacity of bioinspired silica can be estimated from previous studies of amorphous silicas[11], and the same assumptions for the relative power requirements to heat the materials as to maintain heat are used as in the lab-scale calculations (i.e. that the work required to heat the material is 3.75 times the work required to maintain heat).Therefore, given a silica throughput of 40,000 tons and an operating duration of 8000 hours per annum (consistent with our previous work[12]), a furnace throughput of 5 tons per hour and a residence time of 8 hours was estimated using equation 2 to be approximately **2.8MW**.

$$Q_{Furnace} = \left(1 + \frac{\tau}{3.75}\right) \cdot \frac{1}{\eta} \cdot \dot{M} \cdot C_p \cdot \Delta T \qquad (2)$$

Where $Q_{Furnace}$ is the calculated furnace duty, τ is the furnace residence time, η is the fuel efficiency, $\dot{M}$ is the mass flowrate of silica through the system, C_p is the silica heat capacity, and ΔT is the temperature change in the furnace.

Acid treatment

Calculating the energy cost of acid treatment to purify bioinspired silica on a process scale depends on the energy cost of mixing extra acid into the reaction media. To estimate this, a conventional stirred tank was designed using the standard geometries defined in reference [13] as liquid height H = tank diameter D, and stirrer diameter R = D/3. Given the specifics of the small-scale experiments from this study (~1 g/L yield, 10 minute residence time), the working tank volume was calculated to be 875 m^3, corresponding to a tank diameter and height of 10.4m. A stirrer speed of 68 RPM was chosen as a standard AGMA gearbox output, and a power number for a 4 pitched-blade turbine of 1.73 was used as defined in reference [13]. By using equation (3) for power number the duty of the stirrer was calculated to be approximately **735kW**, a significant reduction over the calculated cost of calcination.

$$P = Po \times n^3 \times D^5 \times r \qquad (3)$$

Where P is the stirrer duty, Po is the stirrer power number, n is the stirrer frequency, D is the tank diameter and ρ is the liquid density.

Ethanol reflux

For ethanol reflux, the largest energy requirement arises from the latent heat of the ethanol, which will be in a constant state of boiling and condensing. From lab-scale studies, the relative quantities of ethanol to silica vary between 150ml ethanol:1g silica[14] to 50ml ethanol:1g silica.[15] For the purposes of this study, we used the

midpoint between these reported values of 100ml ethanol:1g silica. This corresponds to approximately 79g ethanol:1g silica.

As before, a silica throughput of 5 tons per hour was used, corresponding to an ethanol flow rate of 394.5 tons of ethanol per hour. Given an ethanol latent heat of 864kJ/kg,[16] the total heat required for vaporisation would be 92.7MW, however as the system is at reflux this energy should all be returned to the system by the immediate condensation of ethanol. Assuming 5% heat losses across the unit operation, an overall duty of **4.64MW** was estimated using equation (4).

$$Q = (1 - \eta) \cdot \dot{M} \cdot R \cdot \lambda \qquad (4)$$

Where Q is the duty of the ethanol boiler, η is the boiler thermal efficiency, $\dot{M}$ is the silica mass flowrate, R is the mass ratio of silica to ethanol solvent, and λ is the ethanol latent heat.

Sensitivity analysis

Obviously the duties calculated in this section rely on a large amount of assumptions at all stages of their calculation, so a sensitivity analysis was carried out to assess the uncertainty within the calculated power values. Each variable used within the calculations was varied by 10% (with the sole exception of stirrer tank number – as this is a discrete quantity, the value range used was 1-3 tanks) and the ramifications upon the calculated duty were calculated, shown in figure S6.

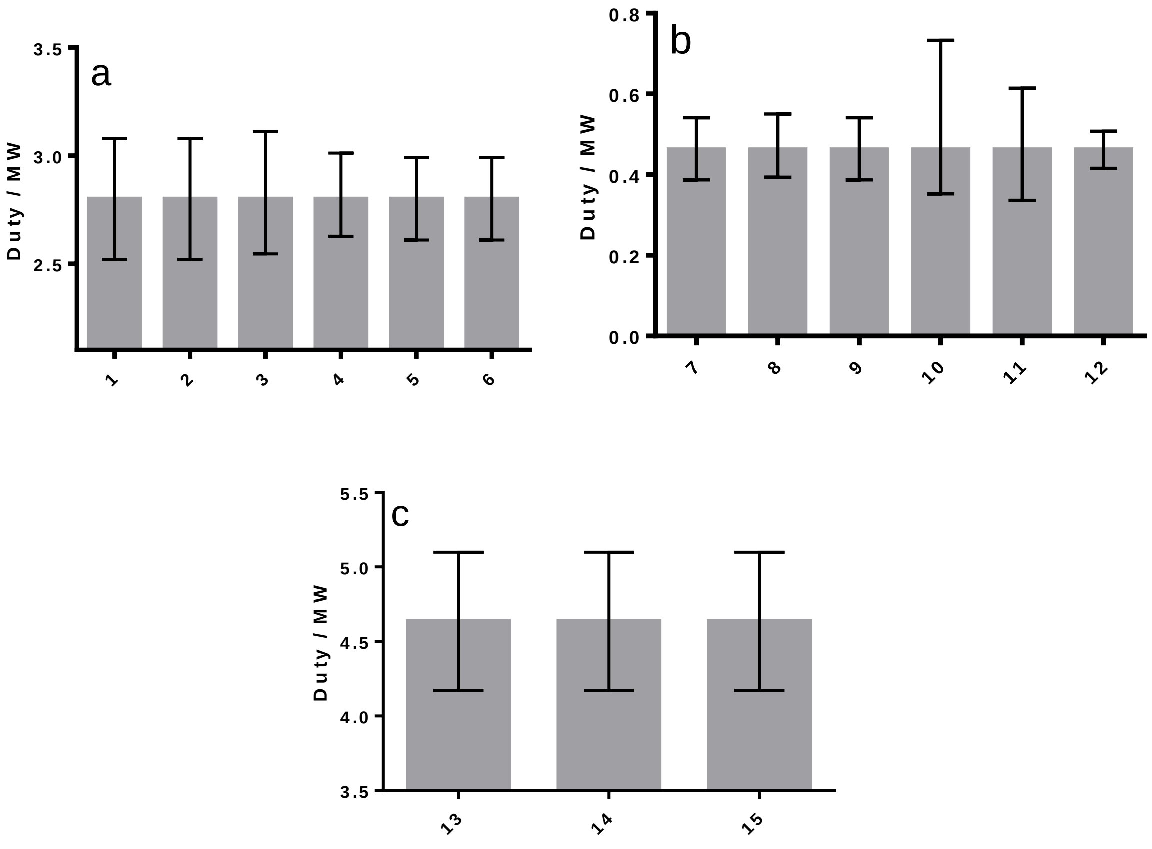

Figure S7. Sensitivity analysis of (a) a continuous furnace, (b) a continuous mixing tank, and (c) continuous ethanol reboiler for calcination, acid treatment, and ethanol extraction of bioinspired silica on an industrial scale, respectively. (1) – Process

volume, (2) – Silica heat capacity, (3) – Furnace fuel efficiency, (4) – Relative power required to heat silica, (5) – Relative power required to maintain silica heat, (6) – Furnace residence time, (7) – Process volume, (8) – Slurry solids loading, (9) – Mixing tank residence time, (10) – Number of mixing tanks*, (11) – Tank stirrer frequency, (12) - Stirrer power number, (13) – process volume, (14) – silica:solvent ratio, and (15) – reboiler thermal efficiency. * Number of mixing tanks was varied from 1 to 3 in parallel.

References for supporting information

[1] K. Milonjic, *Colloids and Surfaces* **1987**, *23*, 301–312.
[2] W. L. Jorgensen, D. S. Maxwell, J. Tirado-Rives, *J. Am. Chem. Soc.* **1996**, *118*, 11225–11236.
[3] H. J. C. Berendsen, J. R. Grigera, T. P. Straatsma, *J. Phys. Chem.* **1987**, *91*, 6269–6271.
[4] H. Heinz, T.-J. Lin, R. Kishore Mishra, F. S. Emami, *Langmuir* **2013**, *29*, 1754–1765.
[5] F. S. Emami, V. Puddu, R. J. Berry, V. Varshney, S. V Patwardhan, C. C. Perry, H. Heinz, *Chem. Mater.* **2014**, *26*, 2647–2658.
[6] B. Hess, C. Kutzner, D. van der Spoel, E. Lindahl, *J. Chem. Theory Comput.* **2008**, *4*, 435–447.
[7] T. Darden, D. York, L. Pedersen, *J. Chem. Phys.* **1993**, *98*, 10089.
[8] J. A. G. Taylor, J. A. Hockey, *J. Phys. Chem.* **1966**, *70*, 2169–2172.
[9] S. H. Hilal, S. W. Karickhoff, L. A. Carreira, *Quant. Struct. Relationships* **1995**, *14*, 348–355.
[10] R. Sinnott, G. Towler, *Coulson and Richardon's Chemical Engineering Volume 6 - Chemical Engineering Design*, **2008**.
[11] J. Horbach, W. Kob, K. Binder, *J. Phys. Chem. B* **1999**, *103*, 4104–4108.
[12] C. Drummond, R. McCann, S. V. Patwardhan, *Chem. Eng. J.* **2014**, *244*, 483–492.
[13] R. K. Grenville, A. T. C. Mak, D. A. R. Brown, A. Products, N. Point, H. Kong, *Chem. Eng. Res. Des.* **2015**, *100*, 282–291.
[14] P. T. Tanev, T. J. Pinnavaia, *Chem. Mater.* **1996**, *8*, 2068–2079.
[15] S. Hitz, R. Prins, *J. Catal.* **1997**, *206*, 194–206.
[16] D. Ambrose, C. H. S. Sprake, R. Townsend, **1975**, 185–190.
